# Flowering Times of Wild *Arabidopsis* Accessions From Across Norway Correlate With Expression Levels of *FT*, *CO*, and *FLC* Genes

**DOI:** 10.3389/fpls.2021.747740

**Published:** 2021-11-01

**Authors:** Hannah Kinmonth-Schultz, Anna Lewandowska-Sabat, Takato Imaizumi, Joy K. Ward, Odd Arne Rognli, Siri Fjellheim

**Affiliations:** ^1^Department of Ecology and Evolutionary Biology, University of Kansas, Lawrence, KS, United States; ^2^Research Support Office, Norwegian University of Life Sciences, Ås, Norway; ^3^Department of Biology, University of Washington, Seattle, WA, United States; ^4^College of Arts and Sciences, Case Western Reserve University, Cleveland, OH, United States; ^5^Faculty of Biosciences, Norwegian University of Life Sciences, Ås, Norway

**Keywords:** *Arabidopsis thaliana*, *CONSTANS (CO)*, *FLOWERING LOCUS C (FLC)*, flowering time, natural variation, *FLOWERING LOCUS T (FT)*

## Abstract

Temperate species often require or flower most rapidly in the long daylengths, or photoperiods, experienced in summer or after prolonged periods of cold temperatures, referred to as vernalization. Yet, even within species, plants vary in the degree of responsiveness to these cues. In *Arabidopsis thaliana*, *CONSTANS* (*CO*) and *FLOWERING LOCUS C* (*FLC*) genes are key to photoperiod and vernalization perception and antagonistically regulate *FLOWERING LOCUS T* (*FT*) to influence the flowering time of the plants. However, it is still an open question as to how these genes vary in their interactions among wild accessions with different flowering behaviors and adapted to different microclimates, yet this knowledge could improve our ability to predict plant responses in variable natural conditions. To assess the relationships among these genes and to flowering time, we exposed 10 winter-annual *Arabidopsis* accessions from throughout Norway, ranging from early to late flowering, along with two summer-annual accessions to 14 weeks of vernalization and either 8- or 19-h photoperiods to mimic Norwegian climate conditions, then assessed gene expression levels 3-, 5-, and 8-days post vernalization. *CO* and *FLC* explained both *FT* levels and flowering time (days) but not rosette leaf number at flowering. The correlation between *FT* and flowering time increased over time. Although vernalization suppresses *FLC*, *FLC* was high in the late-flowering accessions. Across accessions, *FT* was expressed only at low *FLC* levels and did not respond to *CO* in the late-flowering accessions. We proposed that *FT* may only be expressed below a threshold value of *FLC* and demonstrated that these three genes correlated to flowering times across genetically distinct accessions of *Arabidopsis*.

## Introduction

Plants vary both within and across species when it comes to their degree of responsiveness to seasonal changes in daylength (photoperiod) and their complete or facultative requirement for prolonged cold exposure (vernalization) before becoming competent to flower. Presumably, both features have evolved to ensure that plants flower when conditions are most favorable for seed production and maturation (Körner and Basler, 2010; Wang et al., 2014). The model *Arabidopsis thaliana* is a facultative long-day species, which means it flowers most quickly in the long photoperiods during spring and early summer, and can display either a summer- or winter-annual flowering phenotype (e.g., Lee et al., 1993; Gazzani et al., 2003). Those in the former group complete their lifecycle from germination to seed set in a single growing season (spring to summer) and do not require vernalization to flower. Winter annuals germinate in the fall, over winter as a vegetative rosette, and then flower in spring or early summer after a sufficient duration of vernalization, with most accessions either requiring or flowering substantially earlier with vernalization. While we well-understand the mechanisms governing photoperiod and vernalization response in *Arabidopsis*, which has translated to our understanding of photoperiod and vernalization response both in *Brassicaceae* and other species (e.g., Brown et al., 2013; Berry and Dean, 2015; Song et al., 2015; Leijten et al., 2018), we do not yet fully understand the mechanisms governing both intra- and interspecific variation in photoperiod and vernalization responses. Yet, such knowledge is necessary if we are to predict plant responses in dynamic, and changing, natural conditions.

The molecular knowledge gleaned from *Arabidopsis*, coupled with the many wild *Arabidopsis* accessions adapted to different microclimates and displaying variation in their flowering responses to photoperiod and vernalization, provide abundant resources to determine whether there are mechanistic patterns that can be used to predict intraspecific variation, which could be likely applied to other species. In *Arabidopsis*, the molecular and genetic mechanisms controlling photoperiod and vernalization response are well-established in laboratory conditions and some components have been corroborated in the field. *FLOWERING LOCUS T* (*FT*) is a key floral integrator gene, meaning that it is regulated by several upstream genes involved in ambient temperature, vernalization, and photoperiodic sensitivity among other cues (Simpson and Dean, 2002; Capovilla et al., 2015). *FT* is most strongly expressed in long photoperiods and its levels negatively correlate with both the number of days to flowering and with the developmental timing of flowering, i.e., the leaf number at which the reproductive transition occurs, across environmental conditions and mutant lines within a single genetic background (Columbia-0; Col-0) in *Arabidopsis* (Salazar et al., 2009; Krzymuski et al., 2015; Kinmonth-Schultz et al., 2016). *FT* expression is promoted by the *CONSTANS* (*CO*) gene, which peaks in expression during the part of the day that is light in summer and dark in winter to facilitate daylength perception (Suárez-López et al., 2001; Valverde et al., 2004). *FT* is repressed by *FLOWERING LOCUS C* (*FLC*), the attenuation of which is a primary determinant of summer- vs. winter-annual phenotypes (Michaels et al., 2003). In winter-annual variants of *Arabidopsis, FLC* remains high to repress *FT* and delay flowering, until it is epigenetically repressed after a period of vernalization such that winter flowering is inhibited and flowering is promoted in the spring (Searle et al., 2006). *FLC* gene levels and the degree to which it remains repressed after vernalization are often explained by regulatory sequence variation across wild *Arabidopsis* accessions and correlate with flowering time (Li et al., 2014, 2015). In outdoor- and field-grown *A. thaliana* and *Arabidopsis halleri*, *FLC* levels correspond to exogenous temperature, demonstrating the molecular link between environment and phenotypic response (Aikawa et al., 2010; Hepworth et al., 2018).

*FLOWERING LOCUS C* and *FT*, as well as genes upstream of *FLC* and *CO* in the vernalization and photoperiodic pathways, such as *FRIGIDA* and *CRYPTOCHROME 2*, have been frequently associated with natural flowering time variation through quantitative trait loci (QTL)-based approaches yielding valuable insight (e.g., El-Din El-Assal et al., 2001; Li et al., 2006; Schwartz et al., 2009; Sánchez-Bermejo et al., 2012; Méndez-Vigo et al., 2016; Sanchez-Bermejo and Balasubramanian, 2016). However, whether the functional and interactive dynamics of focal genes in these pathways behave as would be predicted from mechanistic laboratory studies (reviewed in Pyo et al., 2014; Song et al., 2015; Perrella et al., 2020) has not been explored across natural populations, although a similar question has been addressed regarding cold tolerance in two *Arabidopsis* accessions native to Italy and Sweden (Gehan et al., 2015). If we could begin to understand how the photoperiod and vernalization pathways interact in wild *Arabidopsis* accessions that are adapted to different microclimates, this information could be used to improve our understanding of plant environmental responses in variable natural conditions.

In earlier work, we described the photoperiod and vernalization responses of several wild *Arabidopsis* accessions collected from throughout Norway and grown together across vernalization periods ranging from 3 to 12 weeks and photoperiods ranging from 8 to 24 h (Lewandowska-Sabat et al., 2012a, 2017). These accessions were collected because the photoperiods varied drastically across collection sites, ranging from about 19 h in southern Norway to 24 h above the Arctic Circle at the summer solstice, and the collection sites were selected to be distant from settlements and roads to avoid introduced populations. These accessions displayed a winter-annual phenotype, either requiring or flowering earlier with vernalization exposure. We found correlative differences in their flowering times and their photoperiod and vernalization responsiveness with altitude (which ranged from 2 to 850 m.a.s.l), distance from the ocean, and microclimate characteristics from their home sites. Briefly, low-altitude accessions, that were closer to the ocean, had overall later flowering and flowered most rapidly after experiencing long photoperiods and prolonged vernalization exposure, while inland, high-altitude accessions flowered rapidly after just 3 weeks of vernalization and were less sensitive to photoperiod (Lewandowska-Sabat et al., 2012a, 2017). It could be possible that variable winter climate and snow cover, at sites experiencing temperature buffering by the ocean, selected for accessions with strong vernalization and photoperiod requirements. Whether the dynamics of key vernalization and photoperiod regulator genes differ across these accessions, and whether those dynamics could explain their flowering differences has not been explored, yet this knowledge would provide insight into the molecular mechanisms controlling flowering along a clear climactic gradient.

We used a subset of 10 winter-annual accessions from our previous work, displaying a range of flowering responses, with eight requiring and two flowering earlier with vernalization, coupled with two well-studied summer-annual lines, Columbia and Landsberg *erecta*, to explore the dynamics of the key photoperiod and vernalization regulators, *CO* and *FLC*, as well as the downstream flowering integrator gene, *FT.* Both Columbia and Landsberg retain summer-annual phenotypes because of null or low functioning alleles of *FRIGIDA*, an upstream activator of *FLC* (Schmalenbach et al., 2014). Landsberg also retains an *FLC* allele with reduced, but not null, function (Koornneef et al., 1994; Michaels and Amasino, 2001; Gazzani et al., 2003). Whether these accessions from Norway, that display a winter-annual phenotype, express high *FLC* levels has not yet been shown. As *FT* levels were shown to be predictive of flowering times in some *Arabidopsis* accessions (Salazar et al., 2009; Krzymuski et al., 2015; Kinmonth-Schultz et al., 2019), and *CO* and *FLC* are important for photoperiod and vernalization response upstream of *FT*, we hypothesized that we would see correlative differences in the expression of these three genes and the flowering phenotypes of these winter-annual accessions native to different locations in Norway. Additionally, since *FT* appears to accumulate over time to influence flowering (Krzymuski et al., 2015; Kinmonth-Schultz et al., 2019), we asked whether expression of these genes would vary temporally over days post vernalization. Finally, since some accessions showed little difference in flowering times between short and long photoperiods when *FT* was typically expressed, we hypothesized that we would find atypically high *FT* expression in these accessions in short days. We demonstrated that the behaviors of these three genes, relative to one another and acting over time, could explain the flowering time behaviors of distinct *Arabidopsis* accessions. This information could likely be used to predict plant responses to dynamic conditions or for plants coming from different environments in the future.

## Materials and Methods

### Plant Material and Growth Conditions

In this experiment, we utilized a subset of previously described *A. thaliana* (L.) Heynh accessions for this work (Lewandowska-Sabat et al., 2010, 2012a,b, 2017). First-generation descendants from individuals collected from 10 locations with diverse climates throughout Norway were used in this study (Supplementary Figure 1 and Supplementary Table 1). These accessions displayed both facultative and obligate vernalization requirements, varying flowering times, and varying responses to long photoperiods, all of which correlated with location and climate variables from the accessions’ homesites (Lewandowska-Sabat et al., 2012a, 2017). Individuals were subjected to 8- and 19-h photoperiods at 16°C after 13 days in 8-h photoperiods at 23°C, and then 14 weeks of vernalization at 4°C and 8-h photoperiods. The long vernalization period was used to saturate the vernalization response. Hydrargyrum quartz iodide (HQI) lighting systems (Osram, Hungary) were used for the 8-h photoperiod as a source of photosynthetically active radiation (PAR), while the 19-h photoperiod was created by adding 1.5 h of light from incandescent bulbs (LU400/XO/T/40 Philips General Electric, Munich, Germany), which have a lower red:far-red ratio, prior to and after a 16-h period using the HQI system to simulate dawn and dusk. Plants were grown in 6.5 cm-diameter soil-filled (Hasselfors Garden AB, Örebro, Sweden) pots and bottomed watered two times per week or once per week during vernalization. For five individuals from each accession, the number of days post-vernalization to the production of a visible bolt (days to bolt; DTB), days to first visible flower (days to flower; DTF), rosette leaf number at flowering, and bolt height at flowering were used for this study and previously described as part of a larger assessment of response to photoperiod (Lewandowska-Sabat et al., 2017). Leaf petiole length, recorded as the average petiole length per plant, and rosette diameter at flowering were also recorded, but have not been previously described. Bolt height data for one accession, and petiole length and rosette diameter for four accessions were not recorded on the same plants used for flowering, and therefore replaced by corresponding data from other descendants from the same parent as the other flowering traits were relatively uniform across descendants from a single parent. Three individuals per treatment of two common lab accessions, Col-0 and Landsberg *erecta*-0 (L*er*-0), were included in this planting for comparison. Data from Col-0 and L*er*-0 has not been previously reported.

### Gene Expression Analysis

Leaf tissue from individuals grown in the 19-h photoperiod and harvested at 1, 9, 13, 17, 20, and 24 h after onset of the PAR, 5 days after being moved from vernalization to the photoperiod treatment, were used for comparison of mRNA accumulation. Tissue from 17 h after PAR onset (zeitgeber 17; ZT17) from days 3, 5, and 8 after vernalization was used to assess gene expression change over time. Tissue was stored at –80°C until processed, moved to 2-ml Safe Seal Microtubes (Sarstedt, Medline, Netherlands) containing three 3.2 mm stainless steel beads (Biospec Products, Fisher Scientific, Norway), cooled in liquid N, then ground using a TissueLyser (Qiagen, Norway). mRNA was isolated using the Illustra RNAspin Mini kit including on-column DNAse treatment (GE Healthcare, Fisher Scientific, Norway), concentrations were determined using a NanoDrop^TM^ 8000 Spectrophotometer (Thermo Fisher Scientific, Norway), mRNA quality was determined using an Automated Electrophoresis Bioanalyzer System (Agilent, Denmark), cDNA was synthesized using Superscript VILO cDNA synthesis kit (Applied Biosystems, Fisher Scientific, Norway), and qPCR was conducted using SYBR Select Master Mix (Applied Biosystems, Fisher Scientific, Norway) on a QuantStudio^TM^ Real-Time PCR system (Thermo Fisher Scientific, Norway). Expression of *CONSTANS* (*CO*), *FLOWERING LOCUS T* (*FT*), and *FLOWERING LOCUS C* (*FLC*) was assessed relative to *ACTIN* and *IPP2* (Sawa et al., 2007) using the ΔCT method. Primers are listed in Supplementary Table 2.

Leaf tissue from the 8-h photoperiod treatment at the 9-h time point was isolated for comparison against the leaf tissue harvested at the 17-h time point in the 19-h photoperiod on day 5 after vernalization. These time points were selected because they were 1 h post, and did not directly coincide with a rapid switch from light to dark (8-h treatment) or from the HQI to incandescent lighting systems (19-h treatment) in our experimental conditions, as well as because they were close to the end of the light period when *FT* should be strongly expressed as its upstream inducer *CO* is degraded in the dark (Kobayashi and Weigel, 2007). In this case, RNA from both photoperiod treatments was isolated using TRIzol^TM^ (Thermo Fisher Scientific, Norway) followed by DNase treatment in solution using the RNase-Free DNase Set (Qiagen, Norway) followed by RNA cleanup using isopropanol. The remaining steps were as above except that *FT* cycle threshold (CT) values were relativized to *IPP2*.

In all cases, three individuals from each accession, separate from those used to record flowering phenotypes, were sampled at each time point for biological replication. Technical duplicates from randomly selected time points were processed for gene expression analysis to ensure uniformity of processing and pipetting. No-template controls were included with all qPCR runs to ensure sample contamination did not occur. For *FT*, some timepoints within replicates, primarily in Lod-1 and Tje-1, yielded undetectable values. As the same samples yielded consistent expression for the four other genes measured, wherein *FT* was overall low in these accessions, we interpreted the expression of *FT* in these samples to be very low and therefore undetectable.

### Statistical Analysis

Correlations among DTB, DTF, leaf number, bolt height, petiole length, and rosette diameter were assessed using the *cor* function in R (method = pearson, version 3.6.3). Differences in DTB across the two photoperiod treatments were assessed using ANOVA (*aov* function in R, version 3.6.3). In the 8-h treatment, the line from Kvi-1 was missing one individual as it had died early in the experiment. These missing values are imputed by averaging the remaining four to ensure a balanced design (Janssen et al., 2010). Flowering times of Col-0 and L*er*-0 were included in plots for visual comparison, but not included in the statistical analyses of flowering times as their sample size differed (three vs. five). However, they were included in any analyses that included gene expression.

To explore relationships between gene expression and flowering phenotypes, we first approximated the total amount of each gene expressed each day by calculating the area under the curve (AUC) for each time course using the *trapz* function in R (*pracma* package, version 3.0.1) (Borchers, 2021). Expression for Kvi-1 was excluded from further analyses as its levels for *CO* were outside the range of all other lines (Supplementary Figure 2), outside those shown for *CO* for that accession in a pilot experiment with other lines (Supplementary Figure 3), and because its leaves were purple and appeared stressed. Then, we assessed the relationship between the flowering phenotypes and *FT*, *CO*, and *FLC* through ANOVA using the *aov* function in base R and the *anova* function to compare different models for final model selection. These tests were done using accession means as the individuals used for gene expression were not the same as those used to assess flowering, although they were from the same grow-up. Assumptions of normality were assessed on the final models using the boxcox power transformation (*boxcox* function, *MASS* package in R) (Box and Cox, 1964; Venables and Ripley, 2002), and the dependent variable was transformed if necessary. As transformations were determined using the fitted values relative to the independent variables in each model, transformations could differ for the same dependent variable across models, and sometimes no transformations were required. Relationships among genes were analyzed in the same manner, except those values for individual plants were used as all five genes were measured in each individual, allowing one-to-one correspondence. In addition to transformations to meet normality assumptions, we also log-linearized *FT* and *FLC* to assess behavioral trends across accessions grouped into different flowering types.

To compare gene expression over time, we grouped the lines by rapid and slow flowering in terms of their DTB and included the lab accessions as a separate group. We then normalized the expression in each group by the maximum value within each accession or strain so that we could compare patterns of change rather than level. Finally, we used ANOVA to compare the effects of time, flowering type (group), and the time by type interaction using the *aov* function in R.

## Results

### Plant Structure Differs With Flowering Time Strategy

**FIGURE 1:**
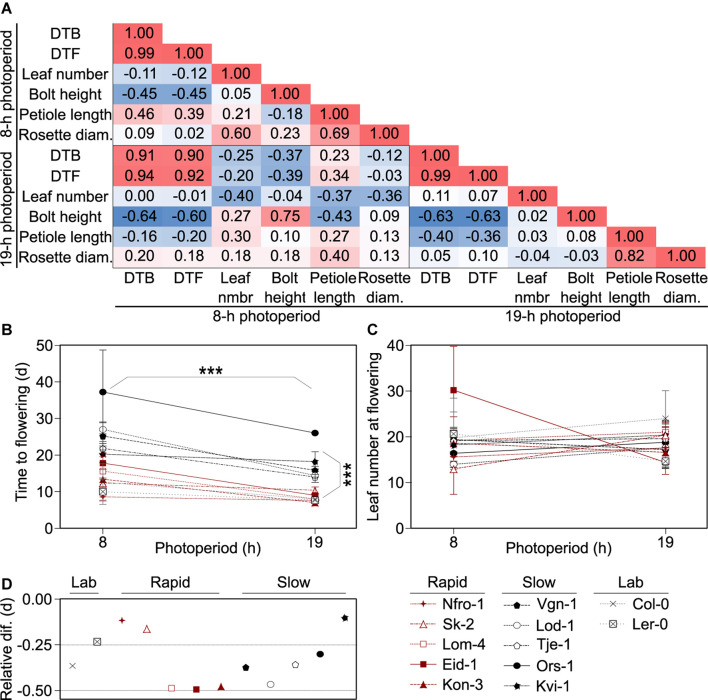
Flowering growth forms and behaviors vary across accessions. **(A)** Pearson correlation matrix showing the correlations between days to bolt (DTB) and days to flowering (DTF) post vernalization, rosette leaf number, flowering stem (bolt) height, leaf petiole length, and rosette diameter at flowering among *Arabidopsis* accessions originating from throughout Norway and grown in 8- or 19-h photoperiods. Red indicates positive correlation and blue indicates negative correlation with the higher intensity hues indicating stronger correlations. **(B,C)** Flowering time for plants grown in 8- and 19-h photoperiods as measured by days post vernalization prior to production of a visible bolt (DTB, **B**) or as final rosette leaf number **(C)**. Asterisks indicate significance between the two photoperiod treatments and flowering types: *rapid-*flowering and *slow*-flowering post vernalization, as indicated by visible separation in the 19-h photoperiod treatments in DTB. Points and lines are color-coded by flowering type: rapid (red), slow (black), or lab (gray). Points represent means of five individuals for each accession and individual, except for those classified as “lab” accessions which represent three individuals. Lab accessions are included in the plot for visual comparison but were not included in the statistical analysis. Error bars are standard deviations. For some points, error bars are smaller than the diameter of the points and therefore not visible. **(D)** Relative change in flowering, calculated as the ratio between the difference in DTB in the 19- and 8-h photoperiods and DTB in the 8-h photoperiods. Across all accessions, flowering is earlier in the 19-h photoperiod relative to the 8-h photoperiod. Those points closer to zero show a smaller difference in DTB between the two treatments. The flowering time data is a subset of data previously reported in Lewandowska-Sabat et al. (2017). Nfro-1 and Lod-1 flower earlier with vernalization but do not require it to flower (Lewandowska-Sabat et al., 2012a).

We first assessed correlations across the phenotypic characteristics in the winter-annual accessions to assess which characteristics we would pursue for further study and to better understand how the plant structure might vary across these wild accessions. We focused on the long photoperiod as the original seeds were collected well after photoperiods exceeded 16 h and plants from these accessions likely flowered in the wild when days were long (Supplementary Table 1). We noted that petiole length and rosette size were correlated, while petiole length and bolt height negatively correlated with the number of DTB (Figure 1A), which meant that later flowering plants were potentially flowering at a smaller size and perhaps had a more compact growth habit than early flowering plants as their petioles were shorter. Leaf number at bolt did not correlate with any other plant trait, even though leaf number is frequently used as a proxy for flowering time in *Arabidopsis* (Pouteau and Albertini, 2009). Plants growing in the short photoperiod had patterns like those in the long photoperiod, except that petiole length positively correlated with DTB. Thus, it is possible that growth form, specifically the compact growth form of the leaves, changes seasonally with daylength in natural settings, but that was not tested here.

Since DTB, DTF, and leaf number are common measures of flowering time (Pouteau and Albertini, 2009), and DTB and DTF were strongly correlated, we moved forward with the comparison of DTB and leaf number, using DTB at it was closer in time to the physiological transition from production of vegetative to reproductive structures. In these winter-annual accessions, we noted two visible groupings in timing to flowering in DTB, especially in the 19-h photoperiod, which were those that flowered more rapidly and those that flowered more slowly post vernalization (Figure 1B). Hereafter, we referred to these groups as rapid flowering type and slow flowering type. This was not dependent on vernalization requirement as Nfro-1 and Lod-1, the two lines facultative for vernalization, fell into different groups (Lewandowska-Sabat et al., 2012a). DTB showed a clear effect due to photoperiod across accessions, while leaf number did not (Figure 1C). We noted that some winter-annual accessions showed little difference across the short and long photoperiod treatments. However, this did not seem to correlate with the flowering type (rapid vs. slow flowering) (Figure 1D).

### Gene Expression of Key Flowering Regulator Genes Correlates With Flowering Time

To assess whether flowering times correlated with gene expression and which measure of flowering time-correlated most strongly, we compared the expression levels of *FT*, *FLC*, and *CO* from leaves harvested at six timepoints on day 5 after vernalization ended and photoperiod treatments began using tissue collected from the long photoperiod treatment. We included two accessions commonly used for lab studies, namely Col-0 and L*er*-0, for which the relationship between *FT* and flowering had previously been shown across treatments (Kobayashi et al., 1999; Corbesier et al., 2007; Kinmonth-Schultz et al., 2016). These accessions were summer annuals, allowing us to assess whether the behaviors in gene expression differed across summer- and winter-annual variants of *Arabidopsis*. *FT* tended to show a two-peak profile across the ten winter-annual Norwegian and two summer-annual lab accessions, showing the highest level at 17 h after onset of photosynthetically active radiation (PAR, zeitgeber 17, ZT17) which occurred at the end of the light period, and a second peak at ZT9 which occurred midway through the 16-h period of PAR light (Supplementary Figure 2). The latter peak is consistent with the morning peak observed in outdoor-grown *Arabidopsis* that was due to a lower red:far-red ratio, which we mimic at dawn and dusk in our conditions (Song et al., 2018). *FLC* showed its highest expression at ZT1, while *CO* tended to peak at the end of the light period. Expression levels of *CO* for accession Kvi-1 were outside the range of all other accessions, showing an atypical mid-day peak, and different from those shown for *CO* for that accession in a pilot experiment (Supplementary Figure 3). We also noted that its leaves were purple and appeared stressed. Thus, we excluded Kvi-1 from further analysis.

Afterward, we assessed whether the expression of *FT*, *FLC*, or *CO* could be predictive of flowering time and whether the two upstream regulators *FLC* and *CO* could be predictive of *FT* expression. To assess flowering time, we used estimates of total expression from day 5 after vernalization, as calculated by determining the AUC for each time course and gene, as independent variables, comparing them against the dependent variables DTB and final rosette leaf number. We used average values across replicates as the plants for which gene expression was measured were destructively harvested and therefore not assessed for flowering, although they were grown with those used for flowering. Since CO protein is degraded in the dark (Valverde et al., 2004), *CO* mRNA produced during the day should have a greater impact on *FT* levels and flowering time. Therefore, we compared eight different models using ANOVA for each measure of flowering time (leaf number and DTB), specifically the three genes with and without their interactions, the three genes including only daytime *CO* expression with and without their interactions, and each of these models including flowering type as an additional variable. The three flowering types were rapid and slow flowering for the winter-annual accessions, as determined from DTB, and summer-annual accessions (hereafter, referred to as “lab” accessions). We sequentially compared nested variants in both groups of models, those including total *CO* and those including only daytime *CO*, to determine which terms to include in the final models.

First, we described the selection process and results for models including DTB as the dependent variable. For the group of models containing total *CO*, no model performed significantly different than the model containing the three genes without interactions, and so neither flowering type nor the interaction terms were included in the final model from that group (Supplementary Table 3). For the group of models containing daytime *CO*, a model containing the three genes without interactions but including flowering type was significantly different from the simplest model form, so it was used for further analysis. Since total *CO* and daytime *CO* describe slightly different biological processes, not different subsets of the same set of independent variables, we report the results of the final models in both groups here (Table 1). For the model containing total *CO*, *FT*, and *FLC*, only *FLC* showed a significant effect on flowering time. While for the model including daytime *CO*, *FT*, *FLC*, and flowering type, *CO* also had a significant effect. *FT* did not have a significant effect on flowering at this time point 5 days after vernalization. Thus, two upstream regulators of *FT* and flowering time, *CO* and *FLC*, likely drive differences in flowering times in these accessions of *Arabidopsis*.

**TABLE 1:** Results of analysis of variance (ANOVA) for days to bolt (DTB) and rosette leaf number (LfNmbr) relative to *FLOWERING LOCUS T* (*FT*), *CONSTANS* (*CO*), *FLOWERING LOCUS C* (*FLC*), and flowering type.

Model	Df	SS	Mean sq.	F	*P*
**DTB ∼ *CO* + *FLC* + *FT***					
*CO*	1	0.08	0.08	0.02	0.893
*FLC*	1	295.05	295.05	70.72	**<*0.0001***
*FT*	1	1.01	1.01	0.24	0.638
Residuals	7	29.20	4.17		
**DTB^0.5^ ∼ *CO*.DAYTIME + *FLC* + *FT* + type**					
*CO*.DAYTIME	1	0.51	0.51	15.42	***0.011***
*FLC*	1	4.37	4.37	131.77	**<*0.0001***
*FT*	1	0.09	0.09	2.69	0.162
Type	2	0.54	0.27	8.07	***0.027***
Residuals	5	0.17	0.03		
**LfNmbr^–0.5^ ∼ *CO* + *FLC* + *FT***					
*CO*	1	1.19e-04	1.19e-04	0.28	0.613
*FLC*	1	8.39e-05	8.39e-05	0.20	0.671
*FT*	1	4.00e-07	4.00e-07	0.00	0.976
Residuals	7	2.98e-03	4.26e-04		
**LfNmbr^–1^ ∼ *CO*.DAYTIME + *FLC* + *FT***					
*CO*.DAYTIME	1	1.18e-05	1.18e-05	0.12	0.738
*FLC*	1	1.28e-05	1.28e-05	0.13	0.727
*FT*	1	5.70e-06	5.70e-06	0.06	0.815
Residuals	7	6.79e-04	9.69e-05		

*Variables transformed as and if indicated to ensure data meet assumptions of normality (*Df* = *Degrees of freedom, SS* = *Sum of squares, alpha* = *0.05).**

*Models are in bold followed by their respective ANOVA tables. Bold, italicized text in column P indicate significant effects.*

For the models including rosette leaf number as the dependent variable, no model including interaction terms or flowering type differed from the simplest models in both model groups (Supplementary Table 3). Therefore, we continued only with the simplest models in each group, and across both models, no term in *FT*, *FLC*, total *CO*, or daytime *CO* showed a significant effect on rosette leaf number (Table 1). These results indicated that the expression of *FT*, *FLC*, and *CO* are not predictive of the total number of leaves produced before flowering in these winter-annual accessions of *Arabidopsis*.

### *CONSTANS* and *FLOWERING LOCUS C* Influence *FLOWERING LOCUS T* Differently Depending on Flowering Type: Summer Annuals, Rapid-, and Slow-Flowering Winter Annuals

**FIGURE 2:**
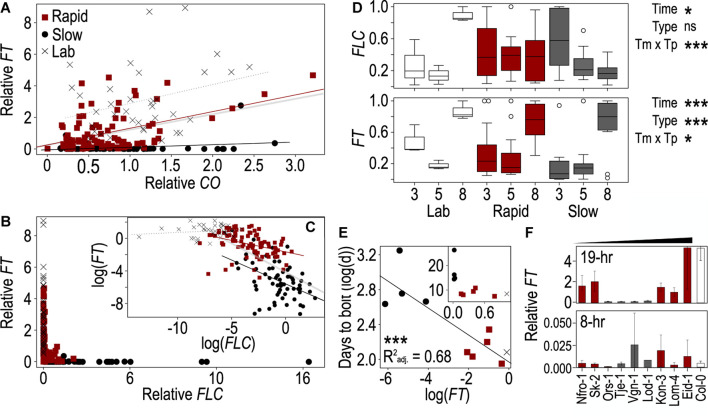
The behaviors of *FLOWERING LOCUS T* (*FT*), *CONSTANS* (CO), and *FLOWERING LOCUS C* (*FLC*) genes vary across accessions and correlate with flowering time. **(A–C)** Correlations between *FT* and *CO* **(A)** and between *FT* and *FLC* **(B,C)** across the *Arabidopsis* accessions used in this study. Lines from winter-annual accessions collected from Norway and classified as rapid flowering (red) or slow flowering (black), while summer-annual “lab” accessions are shown in gray. All points correspond to a single plant grown in one of three biological replicates and harvested at 1, 9, 13, 17, 20, or 24 h after onset of photosynthetically active radiation (PAR, “dawn”). The log-linearized values for *FT* and *FLC* are plotted in panel **(C)**. The thick gray line **(A,C)** indicates the linear trend across all points and accessions, while the other trendlines are specific to flowering type: rapid (red), slow (black), lab (dotted, gray). Values were normalized to the average per replicate before comparing across replicates. **(D)** Change in *FT* and *FLC* expression in plants harvested at 17 h after dawn across days 3, 5, and 8 post vernalization in the rapid, slow, and lab accessions. Relative expression values were normalized to the maximum value within each strain to enable comparison of the change in expression over time. Boxes represent the span between the first and third quartiles, while the middle line represents the median in each group. Asterisks indicate significance of time, flowering type, or their interaction in an ANOVA (*p < 0.05, ***p < 0.0001, ns = not significant). **(E)** Correlation between mean days to bolt for each strain and mean *FT* expression across three biological replicates from plants harvested 17 h after dawn on day 8 after vernalization. Values were log-linearized; inset shows non-transformed data. Asterisks indicate a significance of *p* < 0.0001. Colors indicate rapid, slow, or lab accessions as shown in panel **(A)**. **(F)** *FT* from plants harvested 5 days after vernalization close to the end of the light period 17- and 9-h after dawn and grown in 19- (top) and 8-h (bottom) photoperiods, respectively. The winter-annual accessions are organized by least to greatest difference in DTB between the two treatments. Due to limited resources, only Col-0 was included in panel **(F)**. In all cases **(A–F)**, gene expression was relativized to house-keeping genes prior to any other normalization.

Since *FT* acts downstream of *FLC* and *CO* (Kobayashi and Weigel, 2007), we next assessed the degree to which *FLC* and *CO* were predictive of *FT* levels across these accessions and time points using ANOVA. In this instance, we were able to compare individuals as the values across genes came from the same plants. We compared models including *CO* and *FLC* with and without their interaction as well as with a model including the three flowering types [rapid-flowering winter annuals, slow-flowering winter annuals, and summer annuals (lab)] as a covariate. As the model including the interaction between *CO* and *FLC* and the model including flowering type differed from the model including only *CO* and *FLC*, we included both their interaction and flowering type in the final model (Supplementary Table 4). We found strongly significant effects of *FLC* and a strong interaction between *CO* and *FLC* (Table 2). There was also a significant effect of *CO* which was strongly significant when flowering type was included in the model. To understand how the relationships among these genes varied across the flowering types, we plotted *FT* relative to both *CO* and *FLC* individually across the three groups and assessed the individual of effects *CO* and *FLC* incorporating their interaction with the flowering type (Table 2). The interactions between *FLC* and type and between *CO* and type were both significant as were the individual effects, indicating that each gene affected *FT* as expected and that the effect of each gene on *FT* differed across flowering types. Each flowering type showed very different relationships among the three genes. *FT* showed very low levels across a range of *CO* values in the slow-flowering winter-annual accessions, while *FT* positively correlated with *CO* in both the rapid-flowering winter-annual accessions and summer-annual lab accessions (Figure 2A). The lab accessions showed overall higher levels of *FT* across the same values of *CO* than either the rapid- or slow-flowering winter-annual accessions, but a similar rate of increase to that observed across the rapid-flowering winter-annual accessions although the latter trend appeared driven by a few points. These patterns were the same when replicates were averaged within each accession (Supplementary Figure 4). Together, the data in the rapid-flowering winter-annual accessions and lab accessions were consistent with *CO* as an activator of *FT* and potentially with a scenario in which *CO* activates *FT* at similar rates regardless of flowering type. However, some factor other than *CO* was influencing the overall level of *FT*. Furthermore, *CO* was not predictive of *FT* in the slow-flowering winter-annual accessions at the time point tested, 5 days post-vernalization.

**TABLE 2:** Results of analysis of variance (ANOVA) for *FLOWERING LOCUS T* (*FT*) relative to *CONSTANS* (*CO*), *FLOWERING LOCUS C* (*FLC*), and flowering type.

Model	Df	SS	Mean sq.	F	*P*
Log(*FT*) ∼ *CO* * *FLC* + type					
*CO*	1	29.8	29.8	12.2	***0.0006***
*FLC*	1	345.2	345.2	140.8	**<*0.0001***
Type	2	748.3	748.3	152.6	**<*0.0001***
*CO: FLC*	1	21.4	21.4	8.7	***0.0036***
Residuals	184	451.0	2.5		
*FT* ∼ *CO* * type					
*CO*	1	59.6	59.6	45.4	**<*0.0001***
Type	2	172.0	86.0	65.4	**<*0.0001***
*CO: Type*	2	12.4	6.2	4.7	***0.01***
Residuals	184	241.9	1.3		
*FT* ∼ *FLC* * type					
*FLC*	1	26.76	26.76	18.11	**<*0.0001***
Type	2	176.60	88.30	59.75	**<*0.0001***
*FLC: Type*	2	10.65	5.32	3.60	***0.03***
Residuals	184	271.92	1.48		
Log(*FT*) ∼ Log(*FLC*) * type					
*FLC*	1	790.4	790.4	340.3	**<*0.0001***
Type	2	346.3	173.1	74.5	**<*0.0001***
*FLC: Type*	2	31.6	15.8	6.8	***0.001***
Residuals	184	427.4	2.3		

*Top model variables transformed as indicated to ensure data meet assumptions of normality. The bottom three models coincide with data with and without transformations plotted in Figures 2A–C (*Df* = *Degrees of freedom, SS* = *Sum of squares, alpha* = *0.05).**

*Models are in bold followed by their respective ANOVA tables. Bold, italicized text in column P indicate significant effects.*

The relationship between *FT* and *FLC* showed a different pattern from that of *FT* and *CO*. *FT* was primarily expressed when *FLC* was very low, while *FT* ranged near zero at moderate to high levels of *FLC* (Figure 2B). To better understand the relationship among these genes we log-linearized them both. Doing so revealed different patterns across flowering types. The lab accessions showed no clear relationship between *FT* and *FLC*, likely due to the basal levels of *FLC* expression observed in those accessions as Columbia and Landsberg *erecta* both retain functional variants of *FLC* (Koornneef et al., 1994; Michaels and Amasino, 2001; Gazzani et al., 2003; Schmalenbach et al., 2014). *FT* correlated negatively with *FLC* in the two winter-annual groups. However, the rate at which *FT* decreased with an *FLC* increase was steeper in the slow-flowering winter-annual accessions (Figure 2C). Again, these patterns were the same when replicates were averaged within each accession (Supplementary Figure 4). These data suggested that *FLC* is repressing *FT* with the strongest effect being in the slow-flowering winter-annual accessions. It is likely that the presence of *FLC* is inhibiting *FT* transcriptional activation by *CO* in the slow-flowering winter-annual accessions and influencing overall *FT* levels in the rapid-flowering winter-annual accessions relative to the summer-annual lab accessions. Possibly, there is a threshold level of *FLC* over which *FT* expression is strongly inhibited since the relationship between *FT* and *FLC* across all summer and winter-annual accessions tested was strongly non-linear with *FT* only being expressed above residual levels when *FLC* was low.

### Expression Levels of *FLOWERING LOCUS T* and *FLOWERING LOCUS C* Change Over Time Post Vernalization

While the levels of *CO* mRNA are influenced primarily by the circadian clock and should be relatively stable through time in the constant temperature and daylength conditions used here (Song et al., 2015), *FT* appeared to accumulate over time to influence flowering (Krzymuski et al., 2015; Kinmonth-Schultz et al., 2019), while *FLC* varied temporally post vernalization in accessions of *Arabidopsis* that differ in their strength of vernalization-induced *FLC* repression (Li et al., 2014). Therefore, we hypothesized that the levels of *FLC* and *FT* would change through time post vernalization in these accessions. We compared single time points across days 3, 5, and 8 post vernalization, and to select the time point to use as *FT* and *FLC* peaked at different times of the day, we first determined whether ZT1 or ZT17 from day 5 showed relationships with DTB similar to the full time course. Since ZT17 showed the most similar correlations for both genes (Supplementary Table 5), we selected ZT17 for analysis across the 3 days. We compared values normalized by the maximum value within the three flowering types to allow us to assess change across time rather than the different relative levels among flowering types. We found that *FT* had a strong effect on time and type, as well as a significant interaction between the two terms (Figure 2D). *FT* increased over time across all groups and was highest on day 8. However, the pattern was less clear for the lab accessions, likely contributing to the significant interaction. *FLC* showed a different pattern. There was a slight effect of time and a strong time-by-type interaction. Rapid-flowering winter-annual accessions showed no change over time while slow flowering accessions decreased over time (Figure 2D). The latter was consistent with the *FLC* declines observed post vernalization in some *Arabidopsis* accessions previously (Li et al., 2014). We also assessed the relationship between *FT*, *FLC*, and DTB on days 3, 5, and 8 after vernalization. We noted a strong correlation between *FT* and *FLC* on all 3 days that was strongest on day 3 after vernalization (Supplementary Table 6 and Supplementary Figure 5). Conversely, the relationship between *FT* and DTB was strongest on day 8, while the relationship between *FLC* and DTB was similarly strong across the 3 days (Supplementary Table 6 and Figure 2E). Therefore, the influence of *FT* on flowering likely increased over time, while *FLC* might have retained some influence on *FT* early after vernalization, especially in the slow-flowering winter-annual accessions. A factor other than *FLC* might influence *FT* later.

### *FLOWERING LOCUS T* Levels Cannot Explain the Similarity in Flowering Between Short and Long Photoperiods in Some Accessions

Since we saw little difference in DTB between the short and long photoperiods in some accessions, we hypothesized that *FT* may be more similarly expressed across photoperiods in these accessions. We compared *FT* levels from timepoints near the end of the light periods between plants grown in 8- and 19-h photoperiods as the upstream inducer of *FT*, namely CO protein, was degraded in the dark (Valverde et al., 2004). We found very low levels of expression across all accessions in the 8-h photoperiod (Figure 2F). Within the winter-annual accessions classified as slow, there was little difference between long and short-day treated plants with both treatments having similarly low levels of *FT*. However, rapidly flowering accessions had much higher *FT* levels in long days (Figure 2F). We assessed whether short-day *FT* levels might explain either DTB in short days or the relative change in DTB between the 8 and 19-h photoperiods. However, linear models between *FT* in 8-h photoperiods and DTB in 8-h photoperiods or between *FT* in 8-h photoperiods and the relative change in flowering between 8- and 19-h photoperiods were not significant. Thus, a factor other than *FT* might be contributing to flowering in short days in these accessions.

## Discussion

The relationships among *FT*, *FLC*, and *CO* have been extensively explored such that we now have a solid understanding of how flowering is regulated by photoperiod and vernalization in *Arabidopsis* and this has translated to our understanding in other species (e.g., Brown et al., 2013; Berry and Dean, 2015; Song et al., 2015; Leijten et al., 2018). While natural flowering variation as mediated by variation in *FLC* DNA sequence and expression has been well-described (Bloomer and Dean, 2017), we have little understanding of how these three key flowering regulators differ in their interactions across different wild accessions with different flowering behaviors. We utilized a collection of winter-annual *Arabidopsis* accessions, displaying a range of flowering phenotypes, relative to two well-studied summer-annual accessions to address this question.

Since *FT* is a key floral integrator gene with conserved function across species and a clear relationship to flowering time (Kobayashi et al., 1999; Corbesier et al., 2007; Kobayashi and Weigel, 2007), we expected that the levels of *FT* would correlate to flowering time and this was true for DTB across the nine lines included in this analysis and two laboratory accessions. However, this relationship was strongest on day 8 after vernalization, after *FT* levels had increased in all accessions (Figure 2D). This may indicate that there is a delay post vernalization before *FT* influences flowering time, or it may be that *FT* accumulates overtime before having an effect, possibly through the accumulation of FT protein or by the accumulation of FT-protein-induced changes at the level of downstream genes as *FT* forms a complex with *FD* at the shoot apex to influence flowering transition genes *SUPPRESSOR OF OVEREXPRESSION OF CONSTANS 1 (SOC1)* and *APETALA 1* (Abe et al., 2005; Wigge et al., 2005). Accumulation of FT is consistent with previous studies, in which *FT* was consistent with previous studies, in which *FT* was simulated to accumulate to a threshold value to predict flowering and in which *FT* was induced in single leaves over multiple days (Krzymuski et al., 2015; Kinmonth-Schultz et al., 2019). However, the mode in which *FT* information accumulates is still unknown.

Final rosette leaf number at flowering was not explained by the expression of *FT* contrary to the relationship frequently shown in studies using summer-annual accessions of *Arabidopsis*, which have low levels or weak alleles of *FLC* (Kobayashi et al., 1999; Blazquez et al., 2003; Gazzani et al., 2003; Corbesier et al., 2007; Kinmonth-Schultz et al., 2016). Summer-annual accessions become competent to flower shortly after germination, with *FT* expression beginning by 5 days after germination in long-day conditions, and 11- and 14-day-old plants becoming fully competent to flower after exposure to inductive long-day conditions for 4 and 3 days, respectively (Kobayashi et al., 1999; Corbesier et al., 2007; Kinmonth-Schultz et al., 2016). In this study, the leaf production rate during vernalization likely varied across accessions as was shown across several wild *Arabidopsis* accessions collected along an altitudinal gradient and across recombinant inbred accessions (Méndez-Vigo et al., 2010; Montesinos-Navarro et al., 2011). Additionally, in the summer-annual Col-0 accession of *Arabidopsis*, *FT* was expressed only in leaves produced early after germination, yet this can be predictive of flowering time (Kinmonth-Schultz et al., 2019). Therefore, it is likely that in the winter-annual accessions of *Arabidopsis* described here, we would find a greater correlation between the amount of *FT* measured post vernalization and the number of leaves produced post vernalization. However, we did not separately tally the final leaf number and the number of leaves that had already been produced during the vernalization treatment.

Attenuation of *FLC* expression is a primary factor distinguishing summer- from winter-annual variants of *Arabidopsis* (Michaels et al., 2003). In this study, two distinct winter-annual groups emerged as well, which were also correlated with *FLC* expression. The genetic factors underlying this distinction in flowering “type” are not known. However, *cis*-regulatory variation within the *FLC* locus can dramatically tune flowering times by modulating the rate of placement and temporal stability of epigenetic repressive marks on the *FLC* locus (Shindo et al., 2006; Li et al., 2014, 2015; Yuan et al., 2016; Tian et al., 2019), and *FLC* has been deemed a major factor, not just in response to temperature, but in response to drought and atmospheric carbon dioxide concentrations changes as well (Springer et al., 2008; Dittmar et al., 2014; Fletcher et al., 2016; Méndez-Vigo et al., 2016; Bloomer and Dean, 2017). Changes in flowering time through *FLC* are likely a key avenue through which *Arabidopsis* and other Brassicaceae species adjust to changing conditions through time and space. However, differing threshold requirements of *FT* alleles influences flowering trait variation in *Boechera stricta* (Lee et al., 2014), and variation in *FT*, *FRIGIDA*, upstream of *FLC*, and other climate-responsive genes such as *SHORT VEGETATIVE PHASE* (*SVP*) also influence flowering time in *Arabidopsis* (Stinchcombe et al., 2004, 2005; Schwartz et al., 2009; Méndez-Vigo et al., 2013; Sanchez-Bermejo and Balasubramanian, 2016). Further, phenotypic variation is a result of gene-by-environment interactions as well as of epistatic interactions between genes of interest and the genetic background (Lee et al., 2014), and multiple loci can influence fitness (Price et al., 2018). More work is needed to distinguish the factors underlying flowering type in these populations.

Slow-flowering winter-annual accessions, tending to originate in the low-altitude sites and closer to the ocean (Supplementary Table 1), had the highest levels of *FLC* consistent with our previous proposal that less consistent oceanic climates, which may lack snow cover during parts of the winter, likely necessitate greater flowering repression to inhibit premature flowering in winter (Lewandowska-Sabat et al., 2012a, 2017). In accessions originating from inland, high-altitude sites, *FLC* appears to be repressed more rapidly by vernalization, and it is possible that the consistently cold temperatures caused by consistent snow cover occurring at those sites, mimicked by the consistently cold temperatures during vernalization in our study, serves to repress flowering. If *FLC* is repressed early in these lines, *FT* would be able to be expressed soon after the snow melts in the spring to facilitate rapid flowering. Additionally, flowering times tend to co-vary with other ecologically relevant traits such as germination (Debieu et al., 2013; Takou et al., 2019), and *FLC* and *FT* influence the timing of germination as well (Chiang et al., 2009; Chen et al., 2014). Thus, a broader characterization of the climates at each seed collection site, coupled with analyses of other traits, would be needed to better determine the selective forces driving differences in these accessions.

In the slow-flowering winter-annual accessions, *FLC* appeared to decline over time, while *FT* levels increased in all accessions. *FT* levels negatively correlated with *FLC*, indicating that *FLC* likely contributes to a flowering delay in these lines by repressing *FT* as shown previously (Searle et al., 2006). However, as *FT* was only expressed at the lowest levels of *FLC* across all accessions, it is possible that *FLC* acts somewhat like a switch, strongly blocking *FT* expression until it declines below some threshold level. This concept is similar to how CO protein accumulates at dusk, presumably to some sufficient level, to promote looping of the *FT* chromatin between distal and proximal promoter regions that, then, diurnally disrupts H3K27me3 repressive marks along the *FT* locus (Adrian et al., 2010; Luo et al., 2018; Shibuta and Matsunaga, 2019). Perhaps, in a reverse manner, the presence of FLC protein at the first intron of *FT* (Helliwell et al., 2006) is sufficient to inhibit CO-mediated chromatin looping of *FT* until FLC protein falls below some critical value. If this occurs, a next step would be to determine whether there is natural variation in that threshold level or if natural flowering time variation is driven primarily by initial levels of *FLC* and by its rate of decline with vernalization.

The correlation between *FLC* and *FT* was strongest on day 3 post vernalization, while the correlation between *FT* and flowering was strongest on day 8. This suggests that vernalization through *FLC* influenced *FT* expression early after vernalization, but as *FLC* declined, other factors influenced *FT* later. One factor is CO protein, which appears not to be influencing *FT* expression in the slow flowering accessions studied here, 5 days after vernalization, as *FT* expression did not correlate with *CO* expression in those accessions (Figure 2A and Supplementary Figure 4). However, since CO protein should be consistently expressed in the constant day length and temperature conditions used here (Song et al., 2015), its influence on *FT* should increase as *FLC* declines. Thus, *CO* and *FT* would likely correlate more strongly later in the growing season. Additionally, *FLOWERING LOCUS M* (*FLM*) and *SVP* acted in pathways upstream of *FT* to regulate flowering response to ambient temperature (Lee et al., 2007; Posé et al., 2013; Capovilla et al., 2017). Thus, they might be acting later in the growing season to influence *FT* and flowering time in response to changes in growing season temperatures.

While *FT* production is regulated in the leaves and it is a strong predictor of flowering time (Corbesier et al., 2007), *FLC*, *FLM*, and *SVP* are expressed in and also repress *SOC1* antagonistically with *FT* directly in the shoot apex (Hepworth et al., 2002; Yoo et al., 2005; Helliwell et al., 2006; Lee et al., 2013; Posé et al., 2013). Further, temperature mediates not only *FT* production in the leaves but its rate of transport to the shoot apex (Liu et al., 2020). Thus, there are multiple layers of environmental control in addition to the leaf-level factors assessed here. Finally, some of the winter-annual accessions showed very little difference in DTB between the 8- and 19-h photoperiod treatments that did not correlate with their flowering type (rapid vs. slow), with *FT* levels in the short-day treatments, or the relative difference in *FT* between the short- and long-day treatments. It is possible that *FT* levels increased later in 8-h treatments. However, since *FT* expression in *Arabidopsis* is strongly dependent on long photoperiods (Suárez-López et al., 2001; Valverde et al., 2004), that is less likely. Rather, while *FT* is acting in long days to induce flowering, another factor is likely causing flowering to occur at a similar time in short days. One possible factor is *SOC1* which mediates gibberellic-acid flowering control in short days (Moon et al., 2003). Studies, such as this, that explore the dynamics of genes in known environmental-response pathways across accessions adapted to different climates, will help us determine whether there are predictable patterns of gene activity that can explain observed intraspecific variation in environmental response.

In conclusion, the gene expression behaviors of the key flowering regulator gene *FT* and its upstream regulators *CO* and *FLC* correlated across these wild *Arabidopsis* accessions consistent with the functions of these genes discovered in laboratory settings. In which *CO* and *FLC* were important integrators of the photoperiod and vernalization pathways, respectively. Assessing the behaviors of these genes alone and in conjunction with other layers of molecular flowering control across time and across naturally occurring variants of *Arabidopsis* could help us better understand flowering time regulation in dynamic and natural environments.

## Data Availability Statement

The datasets presented in this study can be found in online repositories. The names of the repository/repositories and accession number(s) can be found below: https://osf.io/jtev8/, Project: Norwegian Accessions –Phenotype x Gene expression.

## Author Contributions

HK-S contributed to the design of the gene extraction experiments, conducted the molecular portions of those experiments, performed the statistical analysis, and wrote the manuscript. AL-S conducted the photoperiod experiments from which the flowering data were extracted, harvested tissue for gene expression analysis, conducted a pilot gene expression analysis, and reviewed the final manuscript. TI advised on the molecular protocols and the manuscript. JW advised on the statistical approach and the manuscript. OAR advised on the design of the plant growth and gene expression experiments and the interpretation of those results. SF conducted the initial seed collections, advised on the design and interpretation of the flowering and gene expression experiments, and contributed to manuscript writing. All authors contributed to the article and approved the submitted version.

## Conflict of Interest

The authors declare that the research was conducted in the absence of any commercial or financial relationships that could be construed as a potential conflict of interest.

## Publisher’s Note

All claims expressed in this article are solely those of the authors and do not necessarily represent those of their affiliated organizations, or those of the publisher, the editors and the reviewers. Any product that may be evaluated in this article, or claim that may be made by its manufacturer, is not guaranteed or endorsed by the publisher.
