# Supplementary material for: Flowering Times of Wild Arabidopsis Accessions From Across Norway Correlate With Expression Levels of FT, CO, and FLC Genes
Source: Front Plant Sci. 2021 Nov 1;12:747740. doi: 10.3389/fpls.2021.747740 (PMC8591261; doi:10.3389/fpls.2021.747740)
Supplement: Supplementary file 1 [file Data_Sheet_1.pdf]

Supplementary Material

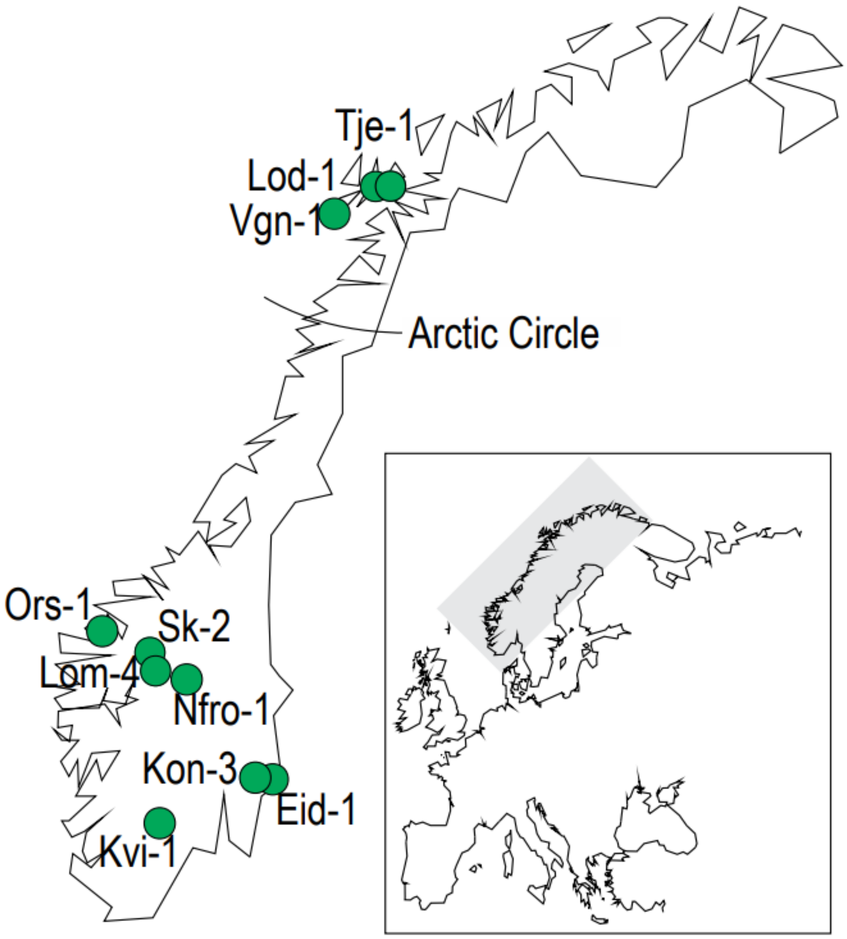

Supplementary Figure 1: Collection sites of *Arabidopsis thaliana* populations used in this study (filled green circles). Norway relative to the rest of Europe is shown (gray box in inset).

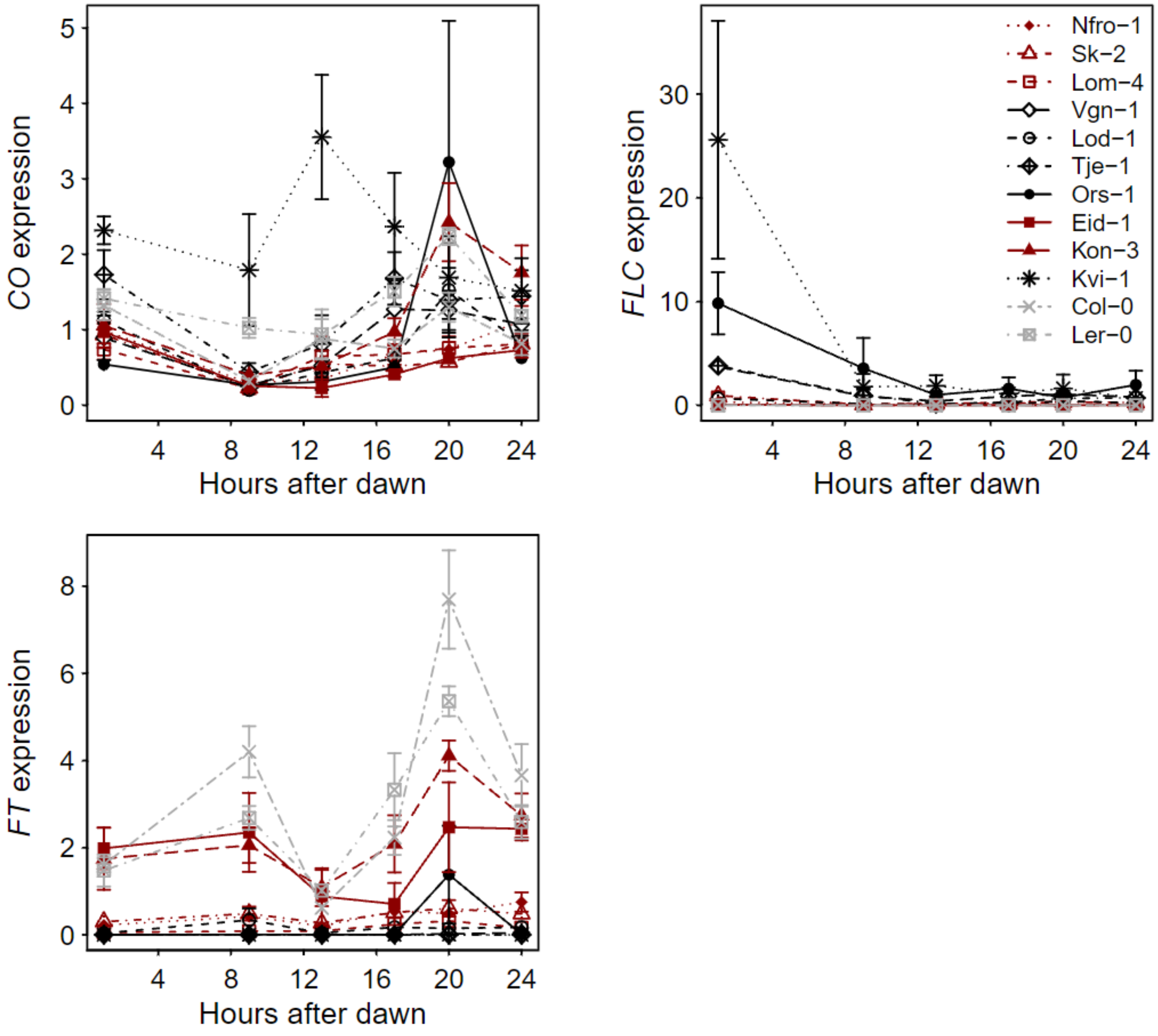

Supplementary Figure 2: Transcript accumulation profiles of *CONSTANS* (*CO*), *FLOWERING LOCUS C* (*FLC*), and *FLOWERING LOCUS T* (*FT*) from plants harvested beginning one hour after onset of photosynthetically active radiation (PAR, 'dawn') five days after vernalization ended and 19-h photoperiod treatments had begun at time points 1, 9, 13, 17, 20, and 24. Red = rapid flowering populations, black = slow flowering populations, grey = lab strains. Transcript accumulation normalized against the average of *IPP2* and *ACTIN* using the ΔCT method, then each replicate was relativized against the replicate mean. Error bars are standard error. Each point represents the average of three biological replicates.

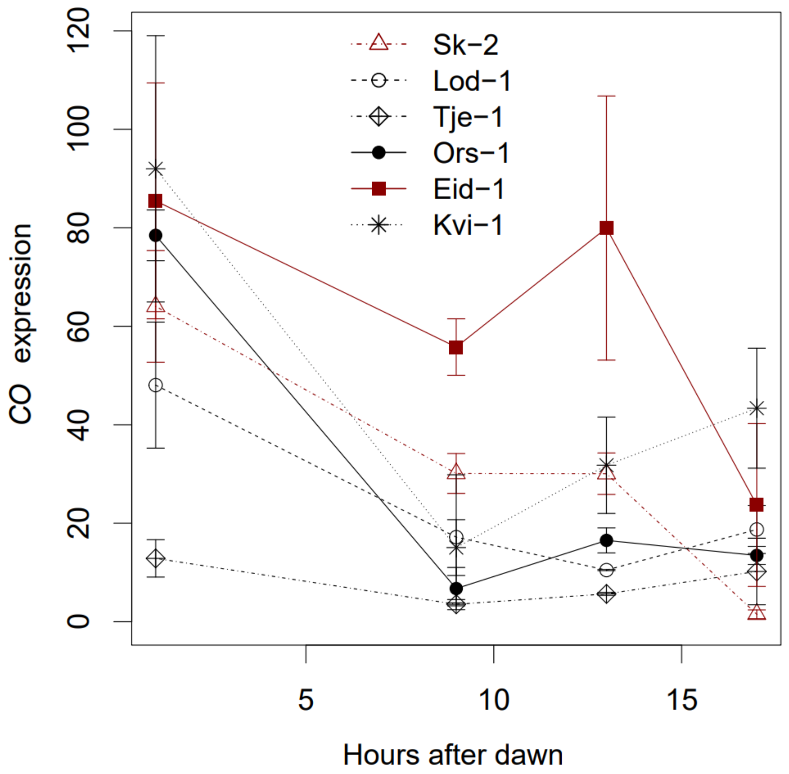

Supplementary Figure 3: Transcript accumulation profile of *CONSTANS* (*CO*) from plants harvested beginning one hour after onset of photosynthetically active radiation (PAR, 'dawn') five days after vernalization ended and 16-h photoperiod treatments had begun at time points 1, 9, 13, and 17. Red = rapid flowering populations, black = slow flowering populations. Transcript accumulation normalized against *ACTIN* using the ΔCT method. Replicates were not relativized as both biological replicates were run on the same plate. Error bars are standard deviation. Each point represents the average of two biological replicates.

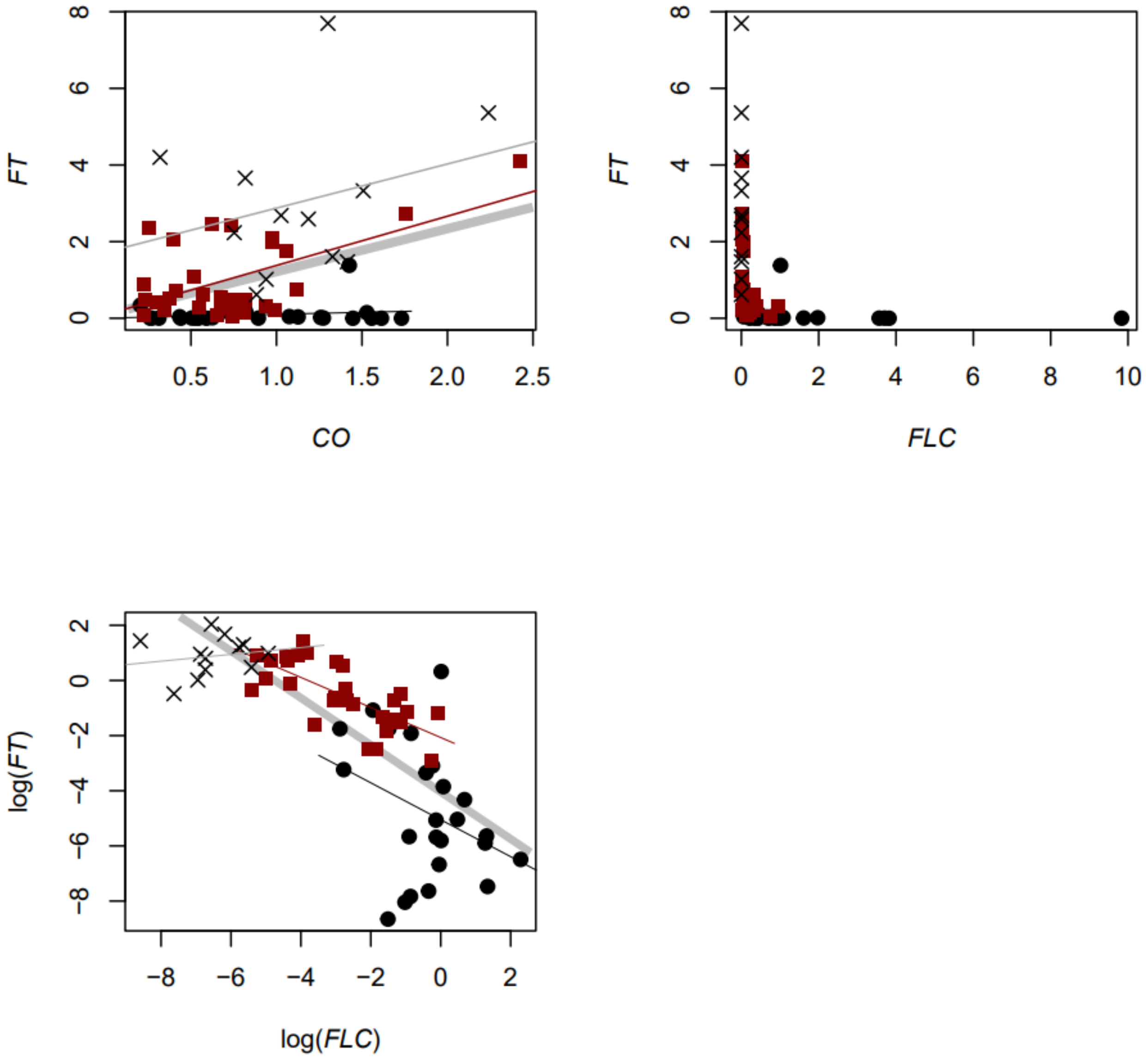

Supplementary Figure 4: Correlations between *FT* and *CO* and between *FT* and *FLC* across the *Arabidopsis* accessions used in this study. Lines from winter-annual accessions collected from Norway and classified as rapid flowering (red) or slow flowering (black), while summer-annual 'lab' accessions are shown in grey. All points correspond to the average of three biological replicates harvested at 1, 9, 13, 17, 20, or 24 hours after onset of photosynthetically active radiation (PAR, 'dawn'). The log-linearized values for *FT* and *FLC* are plotted **(bottom)**. The thick grey line **(left, top and bottom)** indicates the linear trend across all points and accessions, while the other trendlines are specific to flowering type: rapid (red), slow (black), lab (dotted, grey). The values per plant (biological replicate) are shown in Figure 2 in the main text.

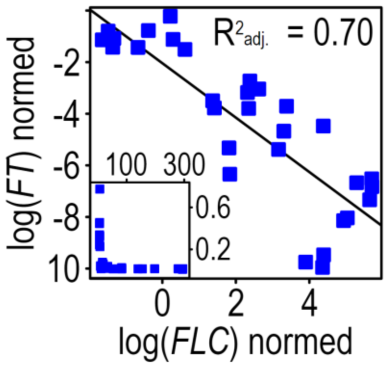

Supplementary Figure 5: Relationship between log-linearized *FT* and *FLC* transcript accumulation in plants harvested 17 hours after onset of photosynthetically active radiation (PAR, 'dawn') three days after vernalization ended and 19-h photoperiod treatments began. Each point represents a single plant from one of three biological replicates after transcript accumulation normalized against the average of *IPP2* and *ACTIN* using the ΔCT method, then each replicate was relativized against the replicate mean. Inset shows non-log-transformed data.

Supplementary Table 1: Population locations, collection dates, and dates photoperiod reached 16 h in spring.

Accession name	Latitude (North)	Longitude (East)	Meters above sea level	Location	Collection date of original population (dd.mm.yyyy)	Dates of Spring 16-h photoperiod (dd.mm)
Lom-4	61°40.880'	08°13.860'	850	Lom	03.07.2003	29.04
Nfro-1*	61°34.647'	09°39.707'	735	Nord-Fron	30.06.2003	29.04
Kvi-1	59°29'	08°26'	703	Kviteseid	09.07.2003	03.05
Sk-2	61°53.307'	08°18.729'	598	Skjåk	02.07.2003	29.04
Kon-3	60°08'	12°05'	300	Kongsvinger	16.06.2003	30.04
Eid-1	60°06'	12°08'	200	Eidskog	16.06.2003	01.05
Tje-1	68°25.498'	16°22.163'	110	Tjeldsund	08.07.2003	18.04
Lod-1*	68°23.851'	15°56.108'	100	Lødingen	08.07.2003	17.04
Vgn-1	68°10.200'	14°13.250'	3	Vågan	07.07.2003	18.04
Ors-1	62°12.330'	06°32.026'	2	Ørsta	15.07.2003	28.04

Photoperiod accessed from www.sunrisesunset.com/custom.asp and recorded as the date at which the light period of each day met or exceeded 16 h. Asterisks indicate populations accelerating with, but not requiring, vernalization to flower.

Supplementary Table 2: Primers for quantitative PCR.

Primer name	Sequence (5' → 3')	Source
F.3 FLC-RT	GGCTAGCCAGATGGAGAATAATCA	NCBI
R.3a FLC-RT	CCGCCGATTTAAGGTGGCTA	
F FT.1-RT	CTGGAACAACCTTTGGCAAT	(Sawa *et al.*, 2007)
R FT.1-RT	TACACTGTTTGCCTGCCAAG	
primerF CO-RT	ATGGCCGGTTCGCAAA	Primer 3
primerR CO-RT	TGTTGTACATTAGCATCGTGTTGAA	
F.2 IPP2-RT	CCCATCGTCCTCTGTCATCG	NCBI
R.2 IPP2-RT	AATGCATTCATCCTCAAACATGAGA	
F ACT-RT	AACTCGAGACAGCCAAAACCA	Primer 3
R ACT-RT	ACGGAATCGCTCTGATCCAAT	
F.4 TFL-RT	ACATTCCCGGCACAACAGAT	NCBI
R.4 TFL-RT	ACACGTCTTTGCTTCTGCCT	

References

Sawa M, Nusinow DA, Kay SA, Imaizumi T. **2007**. FKF1 and GIGANTEA complex formation is required for day-length measurement in *Arabidopsis*. *Science* **318**: 261–265.

Supplementary Table 3: ANOVA comparisons of models for days to bolt (DTB) and rosette leaf number (LfNmbr) relative to *FLOWERING LOCUS T* (*FT*), *CONSTANS* (*CO*), *FLOWERING LOCUS C* (*FLC*), and flowering type.

Model	Res. Df	Res. SS	Df	SS	F	***P***
DTB ~ *CO* + *FLC* + *FT* + type	5	9.07				
DTB ~ *CO* * *FLC* * *FT* + type	1	0.01	4	9.06	161.88	0.059
DTB ~ *CO* + *FLC* + *FT*	7	29.20				
DTB ~ *CO* * *FLC* * *FT*	3	21.84	4	7.37	0.25	0.891
**DTB ~ *CO* + *FLC* + *FT***	7	29.04				
DTB ~ *CO* + *FLC* + *FT* + type	5	9.07	2	20.13	5.55	0.054
DTB ~ *CO.DAYTIME* + *FLC* + *FT* + type	5	7.76				
DTB ~ *CO.DAYTIME* * *FLC* * *FT* + type	1	0.12	4	7.64	15.76	0.187
DTB ~ *CO.DAYTIME* + *FLC* + *FT*	7	29.04				
DTB ~ *CO.DAYTIME* * *FLC* * *FT*	3	28.51	4	0.53	0.01	0.999
DTB ~ *CO.DAYTIME* + *FLC* + *FT*	7	29.04				
**DTB ~ *CO.DAYTIME* + *FLC* + *FT* + type**	5	7.76	2	21.28	6.85	***0.037***
LfNmbr ~ *CO* + *FLC* + *FT* + type	5	60.09				
LfNmbr ~ *CO* * *FLC* * *FT* + type	1	7.42	4	52.67	1.77	0.51
LfNmbr ~ *CO* + *FLC* + *FT*	7	75.20				
LfNmbr ~ *CO* * *FLC* * *FT*	3	65.57	4	9.62	0.11	0.97
**LfNmbr ~ *CO* + *FLC* + *FT***	7	75.20				
LfNmbr ~ *CO* + *FLC* + *FT* + type	5	60.09	2	15.11	0.63	0.57
LfNmbr ~ *CO.DAYTIME* + *FLC* + *FT* + type	5	62.64				
LfNmbr ~ *CO.DAYTIME* * *FLC* * *FT* + type	1	8.32	4	54.33	1.63	0.52
LfNmbr ~ *CO.DAYTIME* + *FLC* + *FT*	7	78.68				
LfNmbr ~ *CO.DAYTIME* * *FLC* * *FT*	3	47.02	4	31.66	0.51	0.74
**LfNmbr ~ *CO.DAYTIME* + *FLC* + *FT***	7	78.68				
LfNmbr ~ *CO.DAYTIME* + *FLC* + *FT* + type	5	62.64	2	16.03	0.64	0.566

Model pairs for each dependent variable sequentially compared. Models selected for final analysis from each group are in bold. Bold, italicized text in column P *indicate significant effects. Res. = Residuals, Df = Degrees of freedom, SS = Sum of squares, alpha = 0.05*

Supplementary Table 4: ANOVA comparisons of models for *FLOWERING LOCUS T* (*FT*) relative to *CONSTANS* (*CO*), *FLOWERING LOCUS C* (*FLC*), and flowering type.

Model	Res. Df	Res. SS	Df	SS	F	*P*
FT ~ CO + FLC + Type	185	254.03				
*FT ~ CO * FLC* + Type	184	245.84	1	8.19	6.12	***0.01***
FT ~ CO + FLC	187	403.07				
*FT ~ CO * FLC*	186	367.48	1	35.59	18.01	***<0.0001***
*FT ~ CO * FLC*	186	367.48				
***FT ~ CO * FLC* + type**	184	245.84	2	121.64	45.52	***<0.0001***

Model pairs for each dependent variable sequentially compared. Model selected for final analysis is in bold. Bold, italicized text in column P *indicate significant effects. Res. = Residuals, Df = Degrees of freedom, SS = Sum of squares, alpha = 0.05*

Supplementary Table 5: Linear correlations between flowering and *FLC* or *FT* from various single time points on day five after vernalization.

Comparison	int.	slope	R^2	$R_{adj.}^2$	*P*
ZT1: log(DTB) ~ log(*FT*)	2.10	-0.11	0.72	0.69	***0.0005***
ZT17: log(DTB) ~ log(*FT*)	2.17	-0.10	0.65	0.61	***0.0016***
ZT24: log(DTB) ~ log(*FT*)	2.21	-0.10	0.59	0.55	***0.0036***
ZT1: DTB ~ *FLC*	10.26	0.5	0.42	0.36	***0.0225***
ZT17: DTB ~ *FLC*	8.56	9.82	0.84	0.82	***<0.0001***

Variables transformed as and if indicated to ensure data meets assumptions of normality. Bold, italicized text in column P indicate significant effects. ZT = hours after onset of photosynthetically active radiation (PAR, 'dawn')

Supplementary Table 6: Linear relationships among *FT*, *FLC*, and DTB.

Comparison	int.	slope	R^2	$R_{adj.}^2$	*P*
Day 3: log(*FT*) ~ log(*FLC*)	-2.04	-1.05	0.71	0.70	***<0.0001***
Day 5: log(*FT*) ~ log(*FLC*)	-2.83	-0.94	0.57	0.55	***<0.0001***
Day 8: log(*FT*) ~ log(*FLC*)	-1.15	-1.19	0.52	0.50	***<0.0001***
Day 3: log(DTB) ~ log(*FT*)	1.95	-0.10	0.55	0.49	***0.014***
Day 5: log(DTB) ~ log(*FT*)	1.95	-0.10	0.56	0.50	***0.013***
Day 8: log(DTB) ~ log(*FT*)	1.99	-0.15	0.72	0.68	***0.0019***
Day 3: DTB ~ *FLC*	8.64	0.06	0.79	0.77	***0.0005***
Day 5: DTB ~ *FLC*	8.64	0.13	0.79	0.77	***0.0006***
Day 8: DTB ~ *FLC*	9.11	0.21	0.83	0.81	***0.0002***

Variables transformed as and if indicated to ensure data meets assumptions of normality. Bold, italicized text in column P indicate significant effects.
